# Microinfusion of Pituitary Adenylate Cyclase-Activating Polypeptide into the Central Nucleus of Amygdala of the Rat Produces a Shift from an Active to Passive Mode of Coping in the Shock-Probe Fear/Defensive Burying Test

**DOI:** 10.1155/2007/79102

**Published:** 2007-06-14

**Authors:** Gabor Legradi, Mahasweta Das, Brian Giunta, Khemraj Hirani, E. Alice Mitchell, David M. Diamond

**Affiliations:** ^1^Department of Pathology and Cell Biology, College of Medicine, University of South Florida, Tampa, FL 33612, USA; ^2^Department of Psychiatry and Behavioral Medicine, College of Medicine, University of South Florida, 3515 East Fletcher Avenue, Tampa, FL 33613, USA; ^3^Department of Psychology, University of South Florida, 4202 E. Fowler Avenue, PCD 4118G, Tampa, FL 33620, USA; ^4^Medical Research Service, Veterans Hospital, 13000 Bruce B. Downs Boulevard, Tampa, FL 33612, USA; ^5^Department of Molecular Pharmacology and Physiology, College of Medicine, University of South Florida, Tampa, FL 33612, USA

## Abstract

High concentrations of pituitary adenylate cyclase-activating polypeptide (PACAP) nerve fibers are present in the central nucleus of amygdala (CeA), a brain region implicated in the control of fear-related behavior. This study evaluated PACAPergic modulation of fear responses at the CeA in male Sprague-Dawley rats. PACAP (50–100 pmol) microinfusion via intra-CeA cannulae produced increases in immobility and time the rats spent withdrawn into a corner opposite to the electrified probe compared to controls in the shock-probe fear/defensive burying test. Shock-probe burying and exploration, numbers of shocks received, locomotion distance, and velocity were all reduced by intra-CeA PACAP injection. Further, intra-CeA PACAP effects were manifested only when the animals were challenged by shock, as intra-CeA PACAP injections did not cause significant changes in the behaviors of unshocked rats. Thus, intra-CeA administration of PACAP produces a distinct reorganization of stress-coping behaviors from active (burying) to passive modes, such as withdrawal and immobility. These findings are potentially significant toward enhancing our understanding of the involvement of PACAP and the CeA in the neural basis of fear and anxiety.

## 1. INTRODUCTION

Pituitary adenylate cyclase-activating polypeptide (PACAP), a member of the secretin/glucagon/vasoactive intestinal peptide superfamily (Arimura and Shioda [1]), is a pleiotropic molecule with remarkable central actions on neuroendocrine and behavioral systems. Intracerebroventricular (icv) or intrahypothalamic PACAP injection results in a significant and long-lasting reduction of food intake (Morley et al. [2]; Chance et al. [3]), elevated plasma vasopressin, mean arterial blood pressure levels, and induces c-fos and vasopressin gene expression in the hypothalamus (Murase et al. [4]; Nomura et al. [5]). A marked increase in steady-state levels of CRH gene expression in the hypothalamic paraventricular nucleus (PVN) was detected after icv PACAP injection, which was blocked by coadministration of a selective PACAP receptor antagonist (Grinevich et al. [6]). PACAP nerve fibers heavily innervate the majority of corticotropin-releasing hormone (CRH) neurons in the PVN (Legradi et al. [7]) and icv PACAP administration under stress-free conditions in freely moving rats increased corticosterone levels and acutely activated PVN CRH neurons (Agarwal et al. [8]), mimicking important aspects of stress activation. Our group reported that PACAP infused into the PVN increased self-grooming behavior and suppressed ongoing exploratory activity (Norrholm et al. [9]). These data support the view that PACAP acts as an excitatory neuropeptide, recapitulating previously demonstrated behavioral effects of electrical and neurochemical PVN activation (Van Erp et al. [10]; Monnikes et al. [11]). Evaluation of time course of PACAP-induced behaviors indicated a cumulative effect of intra-PVN PACAP administration and restraint stress, thereby supporting our hypothesis that PACAP amplifies the effects of stress on behavior (Norrholm et al. [9]).

The influence of PACAP on brain function has also been investigated in learning and memory studies. For example, icv injection of PACAP facilitated the learning, as well as retrieval, of the passive avoidance response (Telegdy and Kokavszky [12]). This finding further highlighted the potential contribution of PACAP to neurobehavioral responses to aversive or threatening stimuli, but its action site could not be determined from their study, further necessitating specific anatomical pharmacologic identification of PACAP target regions. In addition to the neuroendocrine and grooming effects mediated by the hypothalamus, PACAPergic mechanisms in stress responsivity may be processed through the amygdala. The amygdala is viewed as an interface between sensory information and defensive behavioral output, such as manifestations of fear or anxiety (Maren [13]; Davis [14]; LeDoux [15]). Whereas the lateral and basolateral nuclei are responsible for forming the association between fearful and neutral stimuli, perhaps through potentiation of synaptic transmission, the central nucleus (CeA) is implicated in the behavioral and autonomic expressions of fear (LeDoux [16]; Davis [14]). Strikingly high densities of nerve fibers immunoreactive for PACAP have been identified in the central-extended amygdala that includes the central nucleus of the amygdala (CeA) and the lateral part of the bed nucleus of the stria terminalis(Koves et al. [17]; Kivipelto et al. [18]; Piggins et al. [19]; Kozicz et al. [20]; Hannibal [21]). Likewise, medium to high densities of specific PACAP receptor (PAC1-R) expression were detected in CeA (Hashimoto et al. [22]) suggesting local physiologic role for the peptide.

The dense innervation of the amygdala by PACAP nerve fibers clearly indicates that this peptide can exert a strong, but largely unknown, influence on amygdaloid function. The present study, therefore, was designed to explore PACAP's contribution to the regulation of fear behavior, specifically at the level of the central nucleus of amygdala, using the shock-probe fear test. This method was originally developed as the defensive burying paradigm by Treit and coworkers (Treit and Pinel [23]). Findings from Treit's laboratory and others have suggested that either an increased burying response or increased withdrawal from the probe and immobility would be interpreted as qualitatively different expressions of fear behaviors evoked in response to the electrified shock probe. These two basic modes of coping have been viewed as active or passive, according to several investigators (Roozendaal et al. [24]; Treit et al. [25]; Degroot et al. [26]; De Boer and Koolhaas [27]) enabling the evaluation of aversive behaviors by quantitative, as well as qualitative, criteria. Therefore, in the current study we hypothesized that local administration of PACAP into the central amygdala would exert a strong influence on the expression of coping behaviors in rats exposed to the electrified probe.

## 2. MATERIALS AND METHODS

### 2.1. Animals and surgery

Male Sprague-Dawley rats (Taconic Farms, NY, USA), weighing 210–240 upon arrival, were used for the study (total *n* = 58). Animals were pair-housed until cannula implantation surgery and then single-housed in polycarbonate cages and maintained on a 12:12-hour light/dark cycle (lights on at 0700 hours), with food and water available *ad libitum.* Rats were handled daily for a week and habituated to the environmental conditions in the testing room. All procedures were carried out in accordance with the University of South Florida Institutional Animal Care and Use Committee guidelines regarding the care and use of experimental animals. After an initial 1-week acclimation and handling period, rats were anesthetized with Ketamine (90 mg/kg) and Xylazine (10 mg/kg) and a 24-gauge stainless steel guide cannula (Plastics One, Roanoke, Va, USA) was unilaterally implanted into the right central nucleus of amygdala under stereotaxic control (coordinates: 2.4 to 2.6 mm caudal to bregma, 4.4 to 4.5 mm lateral to the midline, and 5.5 to 5.6 mm below the skull surface) through a burr hole in the skull. Cannulae were secured to the skull with three stainless steel anchor screws and cranioplastic cement and temporarily occluded with a dummy cannula. Following surgery, ketoprofen (5 mg/kg) was injected subcutaneously to minimize post-surgical pain and inflammation. Unilateral cannulation of the right amygdala (Huston et al. [28]) was chosen since this side, compared to the left amygdala, has greater involvement in fear conditioning and anxiety responses (Baker and Kim [29]) and unilateral manipulations are surgically less invasive. Experiments were conducted 7-8 days post-surgery and during the light period of the cycle (1000 and 1400 hours).

### 2.2. Shock-probe fear test

For four consecutive days before behavioral experimentation, rats were given mock injections by attaching the guide cannula to an empty injection connector tubing for 2 minutes in their home cage and then the tubing was disconnected and the rats were exposed to the test chamber without the shock probe for 20 minutes. During the pretest session (the day before the experiment) individual rats were given a mock injection, and exposed to the test chamber in presence of an unelectrified shock probe for 20 minutes. Animal behavior was recorded onto digital video files at these pretest sessions.

Rats were randomly assigned into either the control or experimental groups prior to behavioral testing and infused with artificial cerebrospinal fluid (aCSF control) or PACAP (PACAP38; American peptide company, Sunnyvale, Calif, USA) using a BAS bee syringe pump system (West Lafayette, Ind, USA) connected to a 31-gauge internal cannula (Plastics one, outer diameter 0.25 mm, inner diameter 0.125 mm) with 2.5 mm protrusion below the end of the guide cannula to reach the target region. PACAP was diluted in sterile aCSF containing 0.05% bovine serum albumin (Sigma Chemicals, St. Louis, Mo, USA) and administered at a dose of 50 or 100 pmol into CeA in a volume of 0.2 *μ*L over a 30-second period. The internal cannula remained inserted for 1 minute post injection to prevent backflow and to allow for diffusion of the peptide. The internal cannula was then withdrawn and the animal was placed immediately into the shock-probe fear test chamber.

The shock-probe fear test apparatus consisted of a 46.6 × 28 × 26 cm Plexiglas chamber, evenly covered with 5 cm of Tek-fresh odor-absorbent bedding material (Harlan Teklad, Madison, Wis, USA). The shock-probe (8 cm long and 0.8 cm in diameter) was inserted through a hole on one wall of the chamber, 2 cm above the bedding material and helically wrapped with two copper wires through which electric current could be administered. The probe was not electrified until the spontaneously moving rat touched it with its forepaws, at which point the animal received a brief, 2 mA shock from the shock source (precision animal shocker, model H13-15, Coulbourn Instruments, Allentown, Pa, USA), remotely activated by an investigator using a footswitch. The 20-minute test began once the rat received its first shock and the probe remained electrified for the remainder of this period. To determine whether intra-CeA infusion of PACAP, without shocks, would produce alterations in behaviors, a group of rats was subjected to intra-CeA aCSF or PACAP injections and 20-minute exposure to the test chamber in the presence of an unelectrified probe. Animal behavior in the test chamber was recorded onto digital video tape and then saved as MPEG2 digital video files for subsequent observation, scoring, and automated analysis.

### 2.3. Verification of injection sites

Immediately after behavioral testing, animals were deeply anesthetized with Nembutal (90 mg/kg, ip) and perfused transcardially with heparinized saline followed by a solution containing 2% paraformaldehyde and 2.5% acrolein in .1 M phosphate buffer. Standard Nissl staining by cresyl violet and immunolabeling for PACAP were used to evaluate the injection sites. Tissue preparation for immunohistochemistry was performed according to a previously described method (Norrholm et al. [9]). Free floating coronal sections of the forebrain, taken at 30 *μ*m thickness were pretreated with 1% sodium borohydride in distilled water followed by .5% hydrogen peroxide in phosphate-buffered saline and then preincubated in 10% normal horse serum. Sections were incubated for 3 days at 4°C in rabbit anti-PACAP serum (Peninsula Laboratories Inc., San Carlos, Calif, USA) diluted at 1:10,000 followed by sequential incubations in biotinylated donkey anti-rabbit IgG (1:200, Vector, Burlingame, Calif, USA) and the ABC elite kit (1:100, Vector, Burlingame, Calif, USA). Immunoreactivity was visualized with diaminobenzidine (DAB) as chromogen. A total of fifteen animals with missed cannula placements were excluded from statistical analysis. Ten additional animals were excluded for other problems such as bleeding, necrosis, or inadequate spread of synthetic PACAP immunoreactivity.

### 2.4. Analysis of behaviors

 The following behaviors were analyzed from digital video files either by the automated tracking capabilities of Ethovision or counted using the behavior tracker (version 1.5, www.behaviortracker.com), an event-recorder software: (a) locomotion parameters: locomotion distance, defined as the total distance moved in the arena during the test period and mean velocity of locomotion, (b) probe exploration, including a stretched/attend-like posture oriented toward the probe or directly touching or sniffing the probe, (c) immobility, defined as crouching, sitting, or standing still on at least three feet, with the body motionless except for small and slow, lateral scanning movements of the head, (d) zonal preference, defined as time spent in the zone either away from the probe or near the probe, generated by dividing the length of the test chamber into two equal halves, (e) burying parameters: latency to bury, defined as the time between the first shock and the first burying event, duration of time spent on burying the probe such as spraying bedding materials toward or over the probe, the frequency of burying events and the height of bedding material over the probe at the end of session, (f) numbers of contact-induced shocks, (g) rearing time and numbers of rearing events, (h) grooming time and numbers of grooming events. The rats' reactivity to shock was scored according to a four-point scale (Pesold and Treit [30]) where “1” is head or forepaw flinch only, “2” is whole body flinch and/or walking away from the probe, “3” is whole body flinch and running from the probe, and “4” is whole body flinch and jumping (all four paws in the air), followed by running to the opposite end of the chamber (Pesold and Treit [30]; Treit and Pinel [23]). Mean shock reactivity scores were calculated for each rat by summing the shock reactivity scores and dividing them by the total number of shocks received.

All data were expressed as means ± SEM and analyzed by ANOVA, followed by post hoc analysis using the student-newman-keuls multiple comparisons test (SigmaStat 3.0, SPSS Inc., Chicago, Ill, USA). A probability level of *P* < .05 was considered to be statistically significant.

## 3. RESULTS

As indicators of baseline behavior, measures of exploration of the unelectrified probe were evaluated from recordings made during pretest sessions (last habituation session 24 hours before test day, as described in Section 2). No statistically significant differences were found in numbers of probe exploration events and total time spent on probe exploration among sets of rats prior to their placement into the various treatment groups (*P* = .911 and *P* = .854, resp.).

**Figure 1:**
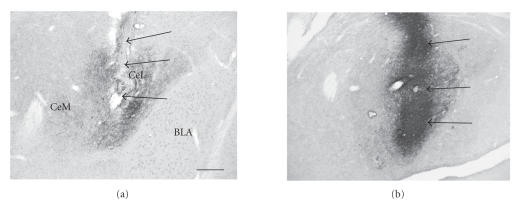
Histological verification of an injection site produced by microinjected synthetic PACAP. (a) Section from control brain, injected with aCSF vehicle. (b) Injected synthetic PACAP (50 pmol) immunoreactivity in the CeA. BLA = basolateral nucleus of the amygdala, CeM = central nucleus of the amygdala medial part, CeL = central nucleus of the amygdala, lateral part. Note the presence of high density of endogenous PACAP fibers in both (a) and (b). Arrows indicate the location of cannula track. In (b), synthetic PACAP injection is visible as an intense dark reaction product. Scale bar = 200 *μ*m.

Figure 1 demonstrates typical injection sites at the level of the CeA using PACAP immunolabeling. The spread of the injected synthetic peptide was verified by the presence of a dense immunoreaction product in addition to the normal appearance of endogenous PACAP nerve fibers (Figure 1(b)).

### 3.1. Effects of intra-CeA PACAP microinjection on probe exploration and zonal preference and locomotion parameters in the rat shock-probe fear test

**Figure 2:**
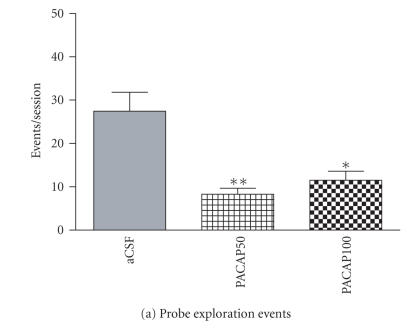

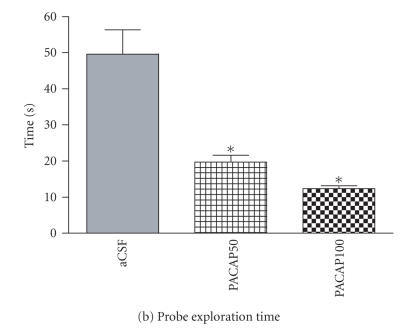

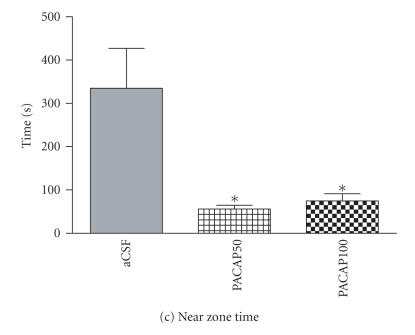

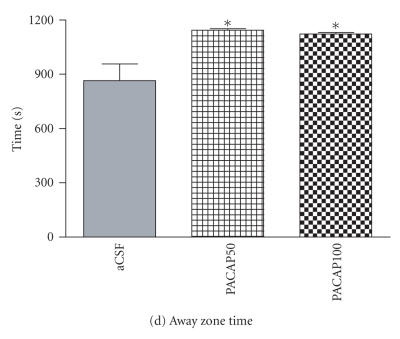
Effects of intra-CeA microinfusion of PACAP on shock-probe exploration and zonal preference in shocked rats. The numbers of probe exploration events (a) and time spent on probe exploration (b) are significantly reduced by intra-CeA PACAP. Zonal preference is altered by intra-CeA PACAP microinjection as rats spent significantly less time in the near zone (c) but more time in the zone away from the electrified shock probe (d). *P < .05 and **P ≤ .001 compared to aCSF controls. (aCSF *n* = 7/group, PACAP50 *n* = 7/group, PACAP100 *n* = 4/group).

One-way ANOVA indicated that PACAP infusion into the CeA significantly decreased the frequency [*F*(1, 9) = 11.05; *P* = .001] and the duration of probe exploration [*F*(1, 4) = 8.15, *P* < .05)] in shocked animals (Figures 2(a), 2(b)). A significant main effect was also found on zonal preference by intra-CeA PACAP microinjection [near zone time; *F*(4, 4) = 6.49, *P* < .05), away zone time; (*F*(4, 4) = 6.52, *P* < .05)] in rats tested with the electrified shock probe (Figures 2(c), 2(d)).

**Figure 3:**
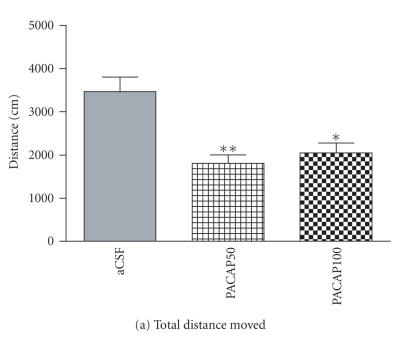

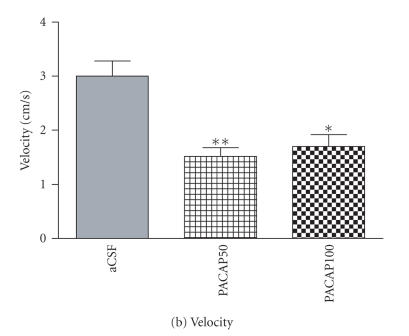

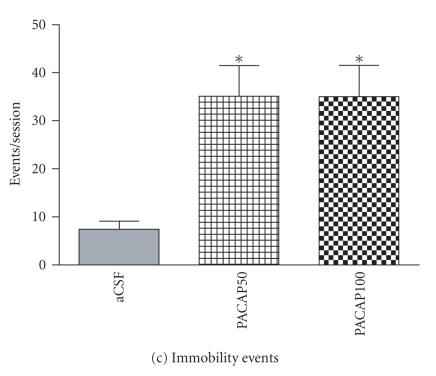

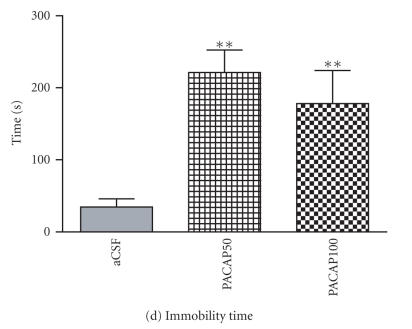
Effects of intra-CeA PACAP microinjection on locomotion parameters in shocked rats. Total distance moved (a) and mean movement velocity (b) were significantly reduced by intra-CeA PACAP microinjection. Immobility events (c) and total time spent on immobility (d) following probe-contact-induced shocks were increased by intra-CeA PACAP. *P < .05 and **P ≤ .001 compared to aCSF controls.

In addition, both total distance moved [*F*(4, 5) = 11.46, *P* < .001] and movement velocity [*F*(3, 5) = 13.11, *P* < .001] were significantly reduced by intra-CeA PACAP injection in shocked groups during the 20-minute test session (Figures 3(a), 3(b)). Immobility behavior was found only in shocked groups, following probe contact-induced shocks. Both the number of immobility events [*F*(7, 5) = 10.49, *P* = .001] and total time spent on immobility behavior [*F*(99, 2) = 226.29, *P* < .001] were significantly increased by intra-CeA PACAP-injection relative to aCSF-injected controls (Figures 3(c), 3(d)).

### 3.2. Burying-related behaviors

**Figure 4:**
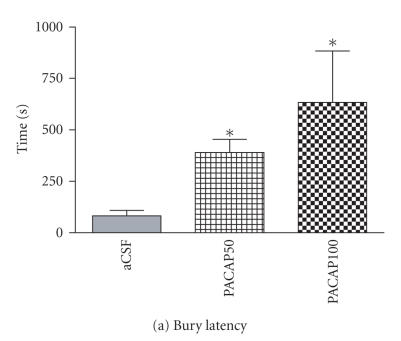

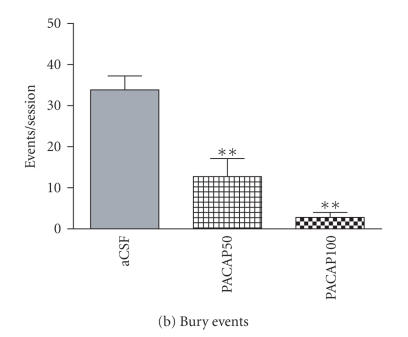

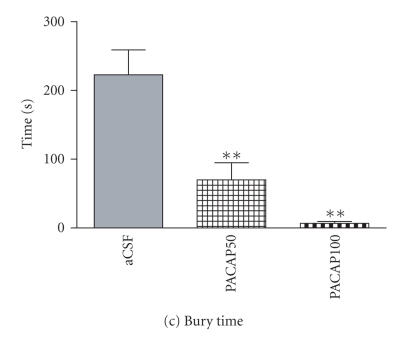

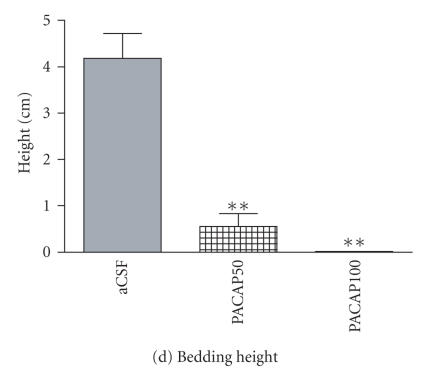

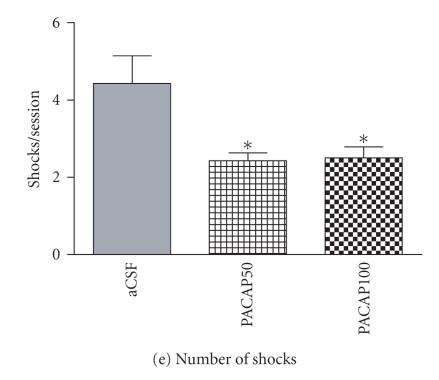

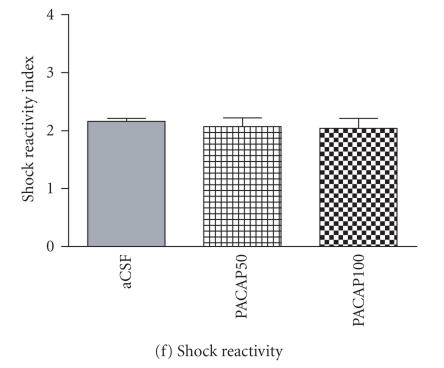
Effect of intra-CeA PACAP administration on shock-probe burying and shock-related behaviors. Latency to bury the electrified shock probe (a) was significantly increased in PACAP-injected animals whereas burying events (b) and time (c) and the height of bedding over the probe (d) were reduced. The number of probe-contact-induced shocks (e) was significantly reduced in PACAP-injected animals but shock reactivity (e) was unaltered. *P < .05 and **P ≤ .001 compared to aCSF controls.

One-way ANOVA indicated a significant main effect of intra-CeA PACAP infusion on bury latency [*F*(4, 4) = 6.55, *P* < .05], total duration of burying [*F*(1, 7) = 13.17; *P* < .001], bury events [*F*(1, 5) = 16.56, *P* < .001], and the height of bedding over the probe [*F*(2, 3) = 31.52, *P* < .001] as compared to aCSF controls. Probe burying was significantly delayed in PACAP-injected rats compared to aCSF controls (Figure 4(a)). Intra-CeA PACAP-injected rats displayed significantly reduced number of burying events (Figure 4(b)). The total amount of time spent on burying the electrified shock probe was also significantly decreased by PACAP injection as compared to aCSF controls (Figure 4(c)). As a result, the height of the bedding material over the probe at the end of the test session was significantly reduced in both the 50 and 100 pmol PACAP-injected groups (Figure 4(d)).

### 3.3. Intra-CeA PACAP infusion reduces number of shocks without altering individual shock reactivity

Intra-CeA PACAP infusion resulted in a significant reduction in the number of shocks received, relative to intra-CeA aCSF-injected rats [*F*(5, 5) = 5.12, *P* < .05] (Figure 4(e)). However, no significant differences were found in the shock reactivity index between aCSF and PACAP-injected groups (Figure 4(f)).

### 3.4. Intra-CeA PACAP injection does not alter exploration of the unelectrified probe or locomotion parameters in unshocked rats

**Figure 5:**
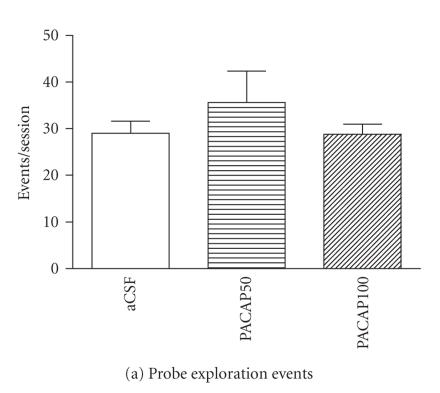

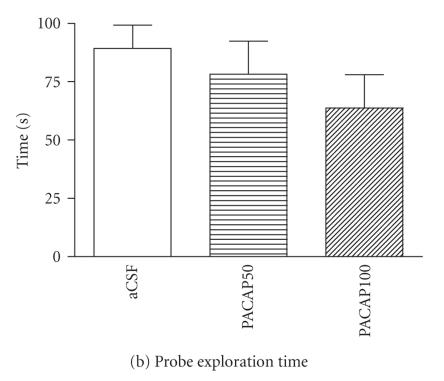

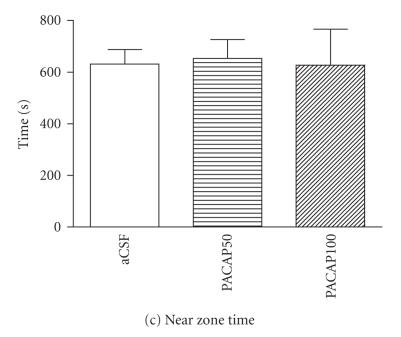

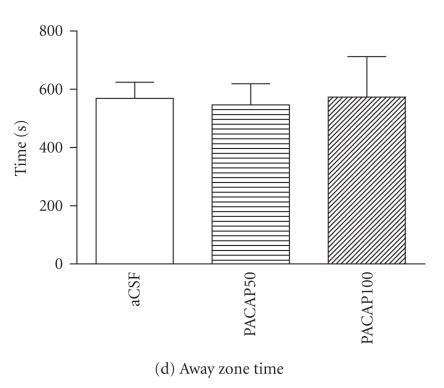

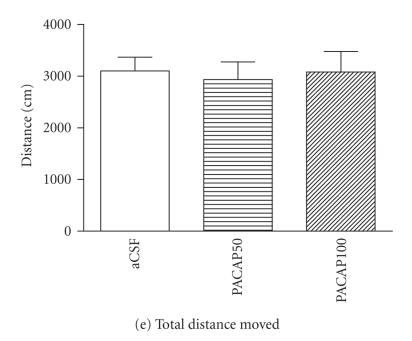

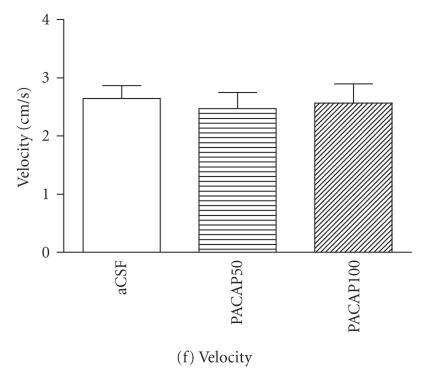
Summary of relevant behaviors of rats tested with the unelectrified shock probe (unshocked groups). Intra-CeA PACAP microinjection, in the absence of shocks, had no significant main effect on rat behaviors in the test chamber. (a) probe exploration events, (b) probe exploration time, (c) near zone time, (d) away zone time, (e) total distance moved, and (f) movement velocity. *P < .05 and **P ≤ .001 compared to aCSF controls. (aCSF *n* = 6/group, PACAP50 *n* = 4/group, PACAP100 *n* = 5/group.)

No statistically significant effects were found in probe exploration in PACAP-injected unshocked groups compared to their respective aCSF-injected controls (Figures 5(a), 5(b)). No intra-CeA PACAP injection effects were found in animals tested with the unelectrified shock probe as in unshocked groups, near and away zone times were roughly equal, and unaltered by intra-CeA PACAP injection (Figures 5(c), 5(d)). In unshocked groups, intra-CeA PACAP injection did not produce statistically significant differences in total distance moved movement or movement velocity compared to their respective aCSF-injected control (Figures 5(e), 5(f)). No burying behavior directed specifically toward the probe was found in unshocked groups, regardless of treatment (data not shown).

### 3.5. Intra-CeA infusion of PACAP does not alter grooming and rearing behaviors in either shocked or unshocked conditions

**Figure 6:**
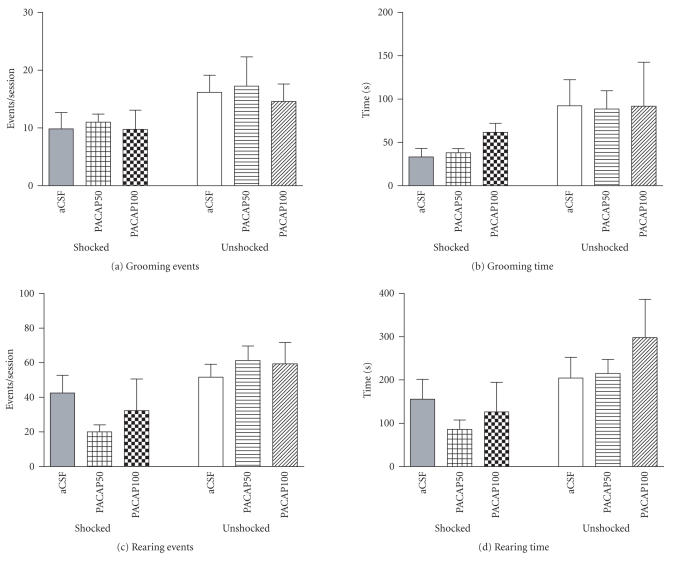
Grooming and rearing behaviors of shocked and unshocked rats in the shock-probe fear chamber. Intra-CeA PACAP microinjection had no statistically significant effects on grooming events (a) and time (b) or rearing events (c) and time (d) in shocked or unshocked groups of rats, relative to their respective aCSF-injected controls.

PACAP microinjection into the CeA at either dose did not significantly alter the frequency or duration of rearing and grooming behaviors as compared to their respective controls (Figure 6).

## 4. DISCUSSION

Since PACAP's discovery, experimental studies have identified roles for PACAP as a multifunctional molecule acting as a neurotransmitter/modulator, neurotrophic factor, supplementary hypophysiotropic hormone, and peripheral vasodilator (Arimura [31]; Vaudry et al. [32]) but the participation of PACAP in neural systems and behavioral functions is inadequately understood. Since strikingly high local concentrations of PACAP immunopositive nerve fibers are found in the central nucleus of the amygdala (CeA) (Koves et al. [17]; Kivipelto et al. [18]; Piggins et al. [19]; Kozicz et al. [20]; Hannibal [21]), a structure associated with the expression of aversion and fear, we hypothesized that PACAP at the level of the CeA could modulate fear-related behaviors. The present study investigated the effects of intra-CeA PACAP microinjection on behavioral responses using the shock-probe fear (defensive burying) test. In this paradigm, the animal is confronted with an electrified shock probe wrapped with uninsulated wires from which shocks are administered. When the spontaneously moving rat touches the probe by exploration, the resultant behavioral response whether active burying or passive (e.g. withdrawal and immobility) can be evaluated using automated and semiautomated observation. In the traditional interpretation of the test, increased probe burying while locomotion is unaltered indicates an anxiogenic response, and reduced burying with increased contact induced shock may indicate anxiolysis. On the other hand, increased withdrawal from the probe and reduction in contact-induced shocks, particularly in the version of the test used by our study where the shock source remains continuously electrified (Treit and Fundytus [33]) can also be interpreted as measures of heightened innate fear. Indeed, our results indicated that intra-CeA microinfusion of PACAP (50 or 100 pmol) enhanced certain types of aversive behaviors in the shock-probe fear test, consistent with our notion that PACAPergic neurotransmission may be linked to manifestations of stress and fear (Agarwal et al. [8]; Norrholm et al. [9]).

In the current study, intra-CeA PACAP injection produced a significant increase in the withdrawal of the shocked rats away from the electrified probe, resulting in dramatically reduced numbers of contact induced shocks. Duration of immobility and time spent in the away zone were markedly elevated in CeA-PACAP-injected animals. Time spent in the near zone, latency of the last shock, duration of burying, and the height of bedding over the probe were also greatly reduced relative to aCSF-injected animals. Measures of locomotion (total distance and time) and velocity of movement were reduced in intra-CeA PACAP-injected animals tested with the electrified shock probe. In the 4-point shock-reactivity scale, (Pesold and Treit [30]; Treit and Pinel [23]), no statistically significant differences were found between intra-CeA vehicle-injected and intra-CeA PACAP-injected rats, indicating that the observed behavioral manifestations were not overtly influenced by organismic variables such as possible changes in shock sensation. Collectively, these data highlight the importance of the CeA in the reorganization of coping strategy in CeA-PACAP-injected animals using the shock-probe fear test to elicit fear and anxiety related responses.

Thus, intra-CeA PACAP-injected animals react with a passive behavioral coping response, which reduces the numbers of shocks received. The mechanisms leading to the behavioral manifestations of PACAP-shock interactions are not known, but we suggest that administration of PACAP in the CeA, likely acting upon its cognate receptor which is widely expressed in the amygdala (Hashimoto et al. [22]), produces its pharmacologic effects locally, on neurons of the CeA. It is therefore possible that the observed pharmacologic effect of PACAP on the behaviors we have described here reflect a role for the endogenous PACAP nerve fibers in the CeA (Koves et al. [17]; Piggins et al. [19]; Hannibal [21]) in the formation of coping behaviors in response to strong aversive stimulation. Determination of the exact contribution of PACAP to responses evoked from the CeA is ultimately dependent on the nature of the target neurons influenced by this neuropeptide. Based on the high concentration of PACAP nerve fibers in the lateral, capsular subnuclei and medium density PACAP innervation in the medial subnucleus of CeA, enkephalin, neurotensin, GABA, and CRH-containing neurons (Cassell et al. [34]) may represent natural targets of PACAP's physiologic effects. Likewise, the behavioral pharmacologic effects observed in the current study most likely reflect PACAP's actions on several classes of CeA neurons that may be interneurons and/or output projection neurons.

It has been recognized that the CeA serves as an output nucleus of the amygdala. Its efferent fibers project to the hypothalamus and brainstem areas such as the periaqueductal gray, parabrachial and caudal pontine reticular nuclei and the nucleus of the solitary tract, which are poised to mediate fear-related behaviors, including immobility and autonomic responses (Hopkins and Holstege [35]; LeDoux et al. [36]; Hitchcock et al. [37]; Saha et al. [38]). Immobility is considered as a first stage of defense when an animal is confronted with a threat, triggering increased vigilance and immobility. In this fear state, the organism has been primed to respond, but is not yet active; an exaggerated startle response is typically found (Lang et al. [39]). CeA lesions block the expression of immobility to fearful stimuli (LeDoux et al. [36]), and attenuate the development of the passive emotional and autonomic components of the coping response (Roozendaal et al. [40, 41]). Activation of the CeA may be linked with the augmentation of passive behavioral coping (Roozendaal et al. [42]) and potentiated startle reflex as well as post-stress freezing (Tinsley and Fanselow [43]).

While the cellular and molecular effects of PACAP have not been examined specifically at the level of CeA, several lines of evidence suggest that in general, PACAP is an excitatory neuropeptide. PACAP is known to colocalize with the major excitatory transmitter glutamate in the retinohypothalamic nerve fibers (Hannibal et al. [44]). The presence of PACAP in primary afferent nerve fibers of the spinal and medullary dorsal horn as well as brainstem cathecholamine neurons also suggests an association with excitatory neurotransmission (Legradi et al. [45]; Dun et al. [46]; Legradi et al. [47]; Das et al. [48]).

Interactions between PACAP and other neuropeptides/neurotransmitters, such as CRH, are quite likely to occur. Based on earlier reports, we hypothesize that the effects of PACAP on fear-related behaviors may be mediated through interaction between PACAP and CRH neurons at hypothalamic, as well as extrahypothalamic, sites (Kozicz et al. [20]; Agarwal et al. [8]). Psychological stress induces CRH gene expression in the amygdala (Makino et al. [49]), antagonism of CRH receptors in the CeA reduces freezing induced by foot shocks (Diamant et al. [50]) and icv CRH administration promotes freezing and reduces shock-probe burying (Swiergiel et al. [51]). Thus, the central action of CRH mediated in part at the level of the CeA is to enhance passive emotional coping. In this context, PACAP in the CeA appears to mimic actions of CRH. Perhaps CRH is an immediate downstream target of PACAP's action in the CeA. If this were the case, then coadministration of a CRH antagonist and PACAP should abolish or significantly blunt the effects of PACAP on fear-related behaviors.

The action of PACAP on the CeA and the resultant reorganization of behavior towards a passive, rather than an active, stress-coping mechanism, is perhaps responsible for shifting of the balance between competing active/passive-coping strategies, regulated by the interplay between various centers of the brain. It is possible that the normally occurring active shock-probe burying response is related to the function of the medial prefrontal cortex (mPFC), a key structure in the organization of goal-oriented behaviors (Haddon and Killcross [52]). The presumed PACAP-induced increase in the activity of the CeA may override the influence of the mPFC (decision-making) process, in favor of the more instinctual immobility responses to shock. In support of this speculation are the findings that mPFC stimulation inhibits CeA output neurons (Quirk et al. [53]), and that excitotoxic lesions of the mPFC or its pharmacologic inactivation with muscimol potently inhibit fear, specifically reducing active stress coping such as shock-probe burying (Shah and Treit [54]; Shah et al. [55]).

It is important to further note that PACAP injection alone, in the presence of an unelectrified probe, did not have an effect on measures of locomotion, immobility, frequency, and duration of probe exploration and zonal preference as compared to the corresponding aCSF- injected controls. Thus, the potentiation of fear-related behaviors by intra-CeA PACAP injection occurred only in shocked rats. This finding provides strong support for the notion that PACAP is active in modifying CeA functions only when the animal is challenged by an aversive stimulus.

In summary, the present study reveals substantial effects of PACAP microinjection into the CeA on the expression of behavioral coping strategies in response to a fear-provoking stimulus. In the shock-probe fear test (defensive burying paradigm), intra-CeA PACAP at 50 or 100 pmol doses induced a remarkable shift from active (burying) to passive (withdrawal) coping strategies. Infusion of PACAP into CeA resulted in no specific alterations in locomotion or probe exploration responses when animals were tested with an unelectrified probe, indicating that PACAP's effects were manifested only when the animal was challenged by aversive stimuli (shock). Thus, in addition to delineating the PACAPergic modulation of amygdala physiology and the neurobiology of fear, these studies may also have important implications toward understanding the role of PACAP in the neural basis of anxiety disorders.
